# AIRRSHIP: simulating human B cell receptor repertoire sequences

**DOI:** 10.1093/bioinformatics/btad365

**Published:** 2023-06-05

**Authors:** Catherine Sutherland, Graeme J M Cowan

**Affiliations:** Institute of Immunology and Infection Research, School of Biological Sciences, University of Edinburgh, Edinburgh, EH9 3FL, United Kingdom; Institute of Immunology and Infection Research, School of Biological Sciences, University of Edinburgh, Edinburgh, EH9 3FL, United Kingdom

## Abstract

**Summary:**

Adaptive Immune Receptor Repertoire Sequencing is a rapidly developing field that has advanced understanding of the role of the adaptive immune system in health and disease. Numerous tools have been developed to analyse the complex data produced by this technique but work to compare their accuracy and reliability has been limited. Thorough, systematic assessment of their performance is dependent on the ability to produce high quality simulated datasets with known ground truth. We have developed AIRRSHIP, a flexible and fast Python package that produces synthetic human B cell receptor sequences. AIRRSHIP uses a comprehensive set of reference data to replicate key mechanisms in the immunoglobulin recombination process, with a particular focus on junctional complexity. Repertoires generated by AIRRSHIP are highly similar to published data and all steps in the sequence generation process are recorded. These data can be used to not only determine the accuracy of repertoire analysis tools but can also, by tuning of the large number of user-controllable parameters, give insight into factors that contribute to inaccuracies in results.

**Availability and implementation:**

AIRRSHIP is implemented in Python. It is available via https://github.com/Cowanlab/airrship and on PyPI at https://pypi.org/project/airrship/. Documentation can be found at https://airrship.readthedocs.io/.

## 1 Introduction

Adaptive Immune Receptor Repertoire Sequencing (AIRR-seq) uses high throughput sequencing to characterize the state and dynamics of B cell receptor (BCR) repertoires and is of increasing use in understanding the molecular basis of immunity and autoimmunity (Yaari and Kleinstein 2015, Zheng *et al.* 2022). BCR repertoire data comprises sequences of the variable regions of BCRs, which are rearranged and hypermutated during B cell maturation. Key to interpretation of this data is the determination of parental VDJ segments for each receptor, as well as identification of nucleotide insertions and deletions, positions of segment junctions, and sequence mutations. Numerous tools have been developed to enable these analyses, with the most commonly used being IgBLAST, IMGT/HighV-QUEST and MiXCR (Brochet et al. 2008, Ye *et al.* 2013, Bolotin *et al.* 2015). Benchmarking of such tools has been limited but work published to date indicates that their outputs can differ, potentially impacting the conclusions drawn from repertoire sequencing experiments (Smakaj *et al.* 2020).

The ability to assess the absolute accuracy of antibody repertoire analysis tools is limited by the lack of experimental datasets where details of all recombination processes are known. Simulation of rearranged BCR data offers an alternative solution for benchmarking where this ground-truth is recorded. Several methods for simulating BCR data have been published (Safonova *et al.* 2015, Ralph and Matsen 2016, Yermanos *et al.* 2017, Marcou *et al.* 2018, Weber *et al.* 2020, Yang *et al.* 2021b; Han *et al.* 2022). However, the usefulness of these tools for benchmarking studies can be limited by their requirements for complex dependencies, nonstandard output file formats, and/or generation of repertoires that are dissimilar to real experimental data. Therefore, we introduce AIRRSHIP (Adaptive Immune Receptor Repertoire Simulation of Human Immunoglobulin Production), a tool for simulating human BCR sequences, which accurately replicates features of experimental data whilst being fast, flexible, and easy to use.

## 2 Implementation

**Figure 1:**
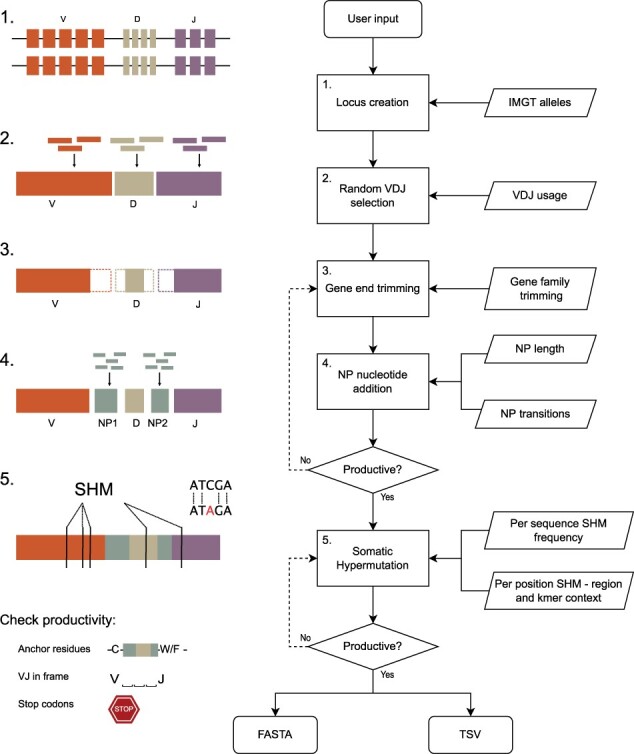
AIRRSHIP simulates human heavy chain BCR sequences, informed by experimental data at each step of the synthetic recombination process. Key parameters such as VDJ usage, gene trimming, junctional insertions and somatic hypermutation can all be modified by the user. Sequences in the FASTA file output can then be used as input for tools of interest and results easily compared to the tab separated values (TSV) file which acts as a record of the recombination process for each sequence.

AIRRSHIP is implemented in Python and has no additional dependencies beyond the standard library. It replicates the processes by which immunoglobulin sequences are formed in vivo (Fig. 1, full details in Supplementary Methods), with each step informed by comprehensive built-in reference data from experimental datasets (Supplementary Tables S1 and S2). To begin, VDJ segments are selected from simulated immunoglobin loci according to observed patterns of usage and an initial recombined sequence is generated. Trimming of segment ends then occurs, with the number of nucleotides to be removed sampled from distributions of trimming lengths for the corresponding IMGT gene family. Following this, NP nucleotides are inserted at the VD and DJ junctions. Insertion lengths are chosen from experimental distributions and addition of nucleotides occurs according to a position dependent Markov process. Separate transition matrices are used when simulating nonmutated and mutated sequences to compensate for the inability to determine which inserted positions have been hypermutated.

Somatic hypermutation is replicated at both the per sequence and per position level. For each sequence, the overall mutation frequency is chosen from a distribution and the required number of mutations are introduced. The likelihood of mutation at each nucleotide is determined by considering the five base sequence motif and immunoglobulin sequence region in which it sits. All reference distributions and models used by AIRRSHIP vary at the IMGT gene, family or sequence region level and thus should be robust to novel allele discovery (Lefranc 2007, Gadala-Maria *et al.* 2015).

To create repertoires tailored to specific benchmarking scenarios, the user can control patterns of VDJ usage, insertions, and deletions, as well as the rate and distribution of somatic hypermutation. Scripts are also provided to allow users to simulate from their own reference data. AIRRSHIP output is optimized for benchmarking, consisting of a FASTA file of sequences which can be used directly as input for tools of interest, as well as a TSV file, which closely follows the AIRR Standards rearrangement schema (Vander Heiden *et al.* 2018) and facilitates easy reference to known sequence origins.

## 3 Results

We assessed the resemblance to real data of sequences generated by AIRRSHIP and three other simulation tools: partis, immuneSIM, and IMPlAntS (Ralph and Matsen 2016, Weber *et al.* 2020, Yang *et al.* 2021b). Key summary statistics from these repertoires were compared to those from published BCR data using sumrep, an R package for comparison and summaries of repertoire data (Olson *et al.* 2019). To prevent favourable bias, experimental data were selected from studies not used for AIRRSHIP input reference (Supplementary Table S3).

Comparison between nonmutated simulated repertoires and IgD/IgM experimental sequences revealed similarly high levels of performance between AIRRSHIP, IMPlAntS, and immuneSIM for VDJ usage frequency. Substantial deviation from experimental data in repertoires generated using partis was observed (Supplementary Fig. S1). Clustering of sequences by usage of VJ gene combinations also indicated that both AIRRSHIP and IMPlAntS produce patterns of recombination that more closely resemble experimental data than immuneSIM or partis (Supplementary Fig. S3). Divergences between real sequences and the AIRRSHIP and IMPlAntS repertoires were similar for lengths of nucleotide deletions and insertions at the junctions, whilst sequences from partis and immuneSIM differed more (Supplementary Fig. S1). The composition of nucleotides inserted at junctions in AIRRSHIP sequences was found to be more similar to experimental data than the insertions produced by any of the other tools. AIRRSHIP uses a Markov process with transition matrices established from human datasets to choose which nucleotide to add at each position of the insertion. In contrast, inserted nucleotides are chosen randomly by IMPlAntS, and immuneSIM selects from NP region and trimming combinations observed from mouse data. The more detailed model used by AIRRSHIP may explain the more realistic insertions produced.

AIRRSHIP repertoires also most closely resembled experimental data in amino acid usage, GRAVY score, aliphatic index, and Atchley factor scores; all of which are important measures of sequence composition. These metrics are measured across the junction region and are representative of the realistic combinations of insertions, deletions, and VDJ gene usage generated by AIRRSHIP. Similar patterns were observed across metrics when comparing simulated repertoires with mutation to experimental IgA/IgG sequences (Supplementary Fig. S2).

During affinity maturation, mutations are introduced unevenly across BCR sequences. Therefore, we compared the ability of the simulation tools to produce realistic mutation patterns (Supplementary Fig. S4). Mutation rates per nucleotide position in the sequence were very closely correlated between AIRRSHIP and experimental sequences (*r* = 0.97). This was comparable to the relationship shown with IMPlAntS repertoires (*r* = 0.95), and stronger than that achieved by partis (*r* = 0.29) and immuneSIM (*r* = 0.27). Mutability models were also inferred using SHazaM (Yaari *et al.* 2013). As with per position mutation rate, IMPlAntS and AIRRSHIP closely replicate experimental patterns, with hotspot and coldspot motifs showing higher and lower mutability, respectively (Supplementary Figs S5 and S6).

Finally, repertoire sequencing datasets may now consist of hundreds of thousands, if not millions, of sequences. Synthetic datasets of similar scale will be required, and these must be able to be produced within a reasonable timeframe. AIRRSHIP can generate 10 000 unique recombinations with mutation in <70 s. This is faster than, but comparable to, IMPlAntS and partis, whilst immuneSIM is considerably slower (Supplementary Fig. S7A and Supplementary Table S5). Sequence generation by AIRRSHIP scales linearly, and one million sequences can be produced in just under 2 h with mutation, or in 13 min without (Supplementary Table S7). When benchmarking, the time taken to achieve the requested number of independent VDJ recombination processes was measured. For IMPlAntS and partis, this can result in final repertoires of greater size when simulating with SHM. Depending on the end goal of the user, in some cases it may be faster than reported for these tools to generate the required repertoire size. Maximum RAM usage was found to be similar for all tools and is unlikely to be prohibitive (Supplementary Table S6 and Supplementary Fig. S7B). All tools were run on an Intel (R) Xeon (R) E5-2407 CPU with eight total cores and 188 GB RAM.

## 4 Demonstration

To illustrate a potential application of AIRRSHIP, we compared the ability of IMGT/HighV-QUEST and IgBLAST to accurately identify the number of nucleotides inserted at the VD junction (Supplementary Fig. S9). Establishing correct gene boundaries within the junction can be complex. During the recombination process, D genes, which are already shorter than V or J genes, may be extensively trimmed such that the remaining sequence has an equal likelihood of having come from multiple parental genes (Yaari and Kleinstein 2015). Distinguishing between mutations and nontemplated nucleotides can also be problematic.

Three repertoires of 100 000 sequences were simulated using AIRRSHIP with default settings. The percentage of sequences where the assigned VD insertion length, as given by IgBLAST or IMGT/HighV-QUEST, matched the number of nucleotides known to be inserted by AIRRSHIP was calculated. Only 49% of all sequences had the correct insertion length assigned by both tools (Supplementary Fig. S9A). The length of the D gene was also shorter in sequences with an incorrect insertion length assignment (9.0 bases versus 14.5 bases, Supplementary Fig. S9B). As trimming of gene ends will result in shorter D genes, simulated repertoires were then generated where the ends of the D gene were not trimmed or where no gene ends were trimmed. In sequences where the D gene was not trimmed, the proportion of sequences with correct assignments was 88%, rising to 97% when no gene ends were trimmed. In our analyses, incorrect assignments will include cases in which inserted nucleotides are identical to those trimmed from the gene ends. It may be impossible for any tool to correctly establish recombination events in these situations but, as they may occur in real sequences, they should not be ignored.

Although identifying differences in performance between the tools themselves was not our primary intention, we did observe that the percentage of sequences where the VD insertion length was correctly identified by both tools was lower than that of each individual tool. This suggests that different sequences are incorrectly analysed by each tool. Overall, gene trimming appears to be a substantial confounder in boundary assignment. By further iterating through AIRRSHIP parameters, a greater understanding of limitations in junctional boundary identification and how these might affect downstream analyses could be gained.

## 5 Discussion

AIRRSHIP simulates BCR repertoires that closely resemble experimental datasets and, for certain metrics, appear more realistic than those produced by other simulation tools. Several factors may contribute to this improved performance. Firstly, all tools were run as presented ‘out of the box’, without providing user data to learn from. In the case of partis, this involved using the *–simulate-from-scratch* method which uses ‘plausible heuristics’ to generate repertoire features rather than replicating a specific sample. This may have contributed to the dissimilarity between partis and experimental sequences. In particular, the SHM model used in this mode does not distinguish between positions in the sequence, explaining the lack of variation in per position mutation rate observed. Partis can also simulate sequences using parameters inferred from user provided data. However, this requires substantially more user input and is therefore not comparable to how AIRRSHIP and the other tools were run.

The choice of in-built reference data may also affect the quality of sequences produced. Data from 380 individual repertoires from five independent datasets, comprising a total of 11 869 126 BCR sequences were processed to create AIRRSHIP reference files. The breadth of this reference data captures variation amongst individuals and the depth should allow even rare BCR sequences to be replicated. In comparison, the majority of immuneSIM parameters are inferred from a single dataset (DeWitt *et al.* 2016) and as such will be limited to features present in these individuals. For many metrics, IMPlAntS performs similarly to AIRRSHIP and this tool also uses reference data from a wide range of experimental datasets, encompassing 2152 individuals (Yang *et al.* 2021a). The differences observed between these tools may be due to the underlying algorithms employed as previously discussed in the case of nucleotide insertion.

As well as producing high quality sequences, AIRRSHIP is designed for ease of use in the production of repertoires for benchmarking applications. It requires few dependencies, runs quickly, and offers broad choices in parameter selection. We have illustrated how AIRRSHIP may be used to explore challenges in AIRR-seq analyses and believe it can be of value in more systematic assessment of the current AIRR-seq tool landscape.

## Supplementary Material

btad365_Supplementary_Data

## Data Availability

AIRRSHIP source code is publicly available at https://github.com/Cowanlab/airrship. This publication makes use of a number of publicly available datasets that are listed in the Supplementary Methods.
