# Supplementary material for: AIRRSHIP: simulating human B cell receptor repertoire sequences
Source: Bioinformatics. 2023 Jun 5;39(6):btad365. doi: 10.1093/bioinformatics/btad365 (PMC10272706; doi:10.1093/bioinformatics/btad365)
Supplement: btad365_Supplementary_Data [file btad365_supplementary_data.zip › Supplementary Figures.pdf]

AIRRSHIP: simulating human B cell receptor repertoire sequences

Supplementary Figures

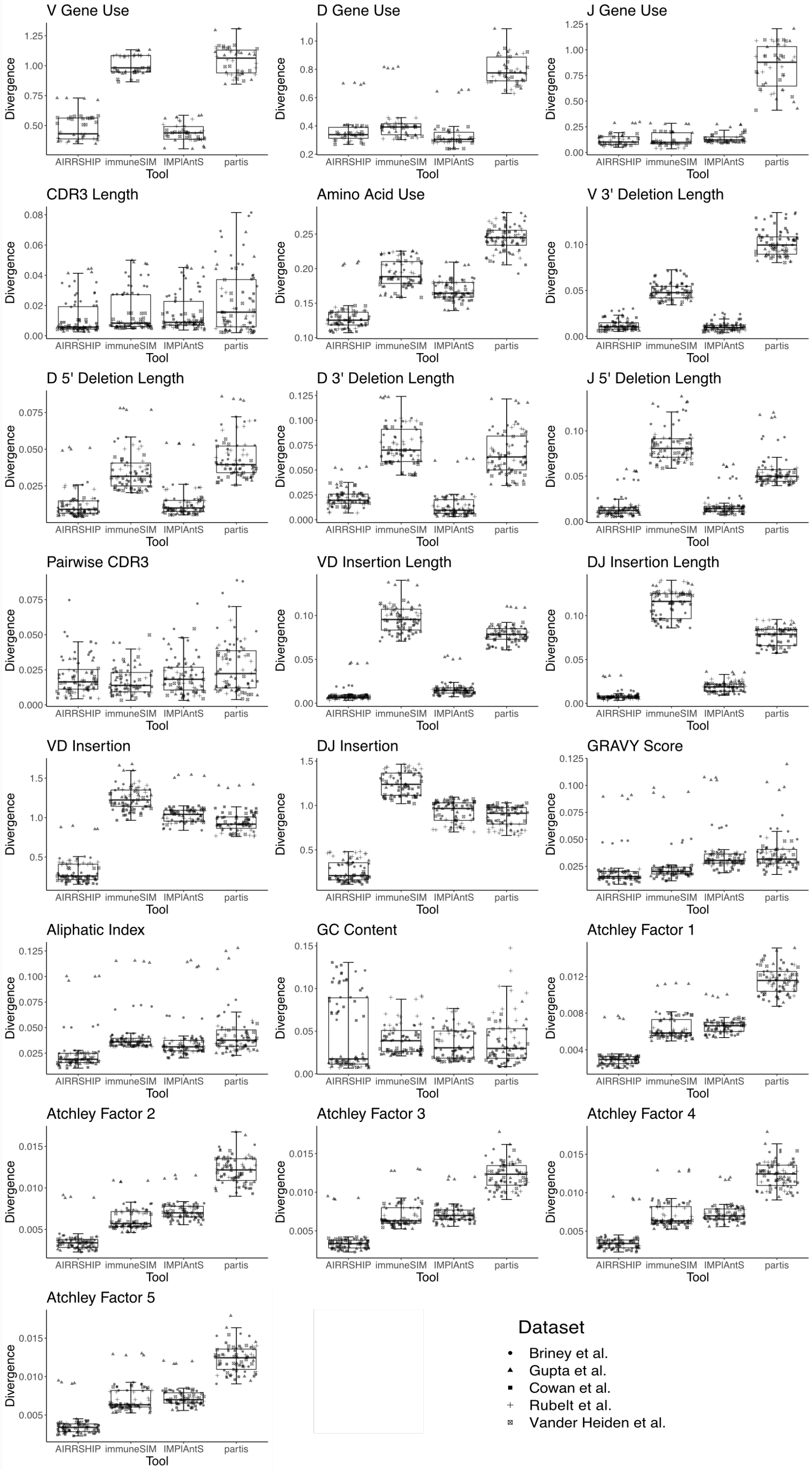

Supplementary Figure S1: Plots of summary divergence values calculated using sumrep – unmutated simulations and IgD/IgM comparisons. For each tool, four simulated repertoires were each compared pairwise to a set of experimental repertoires. Shape of points corresponds to the dataset from which the comparison experimental repertoire originated.

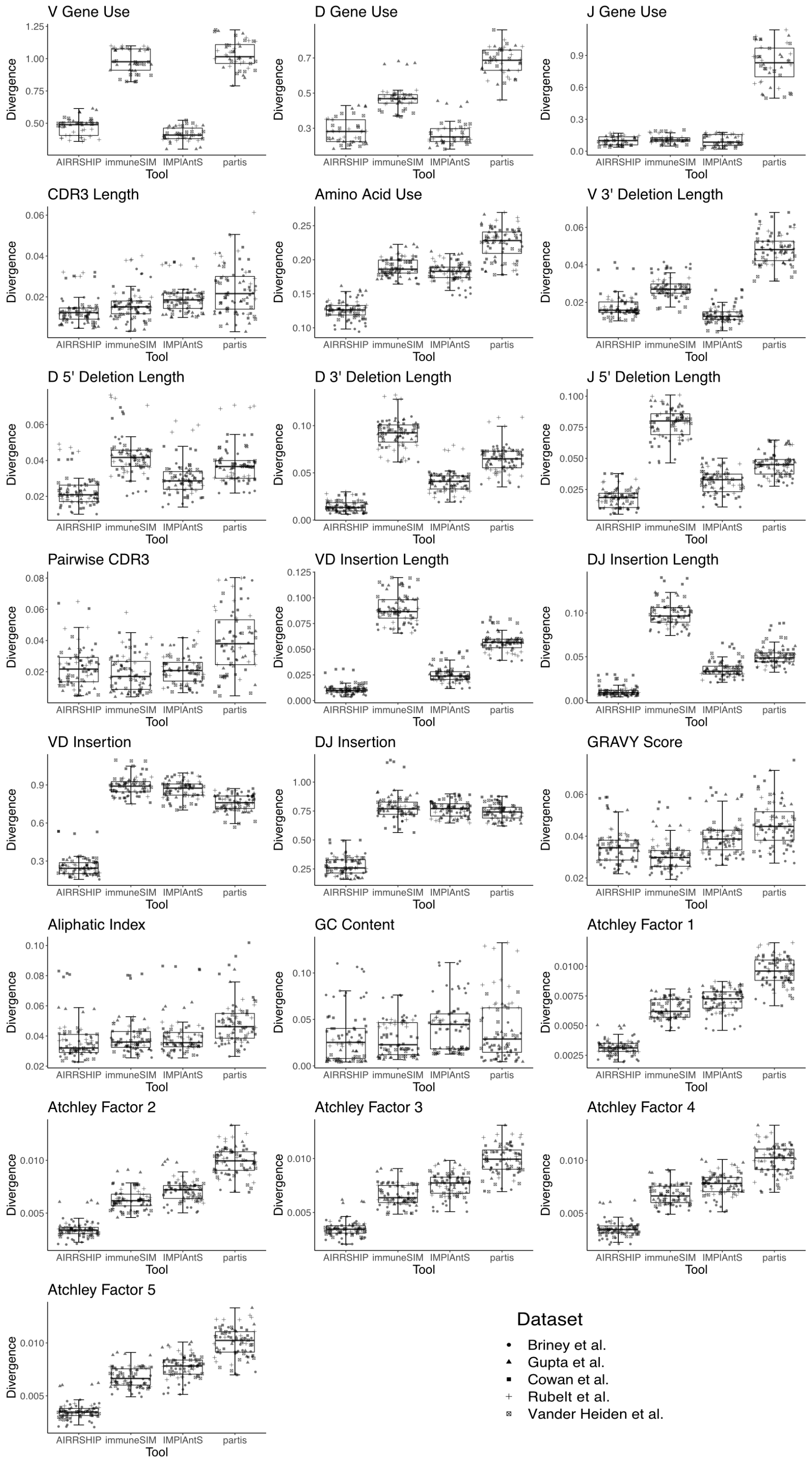

Supplementary Figure S2: Plots of summary divergence values calculated using sumrep – mutated simulations and IgA/IgG comparisons. For each tool, four simulated repertoires were each compared pairwise to a set of experimental repertoires. Shape of points corresponds to the dataset from which the comparison experimental repertoire originated.

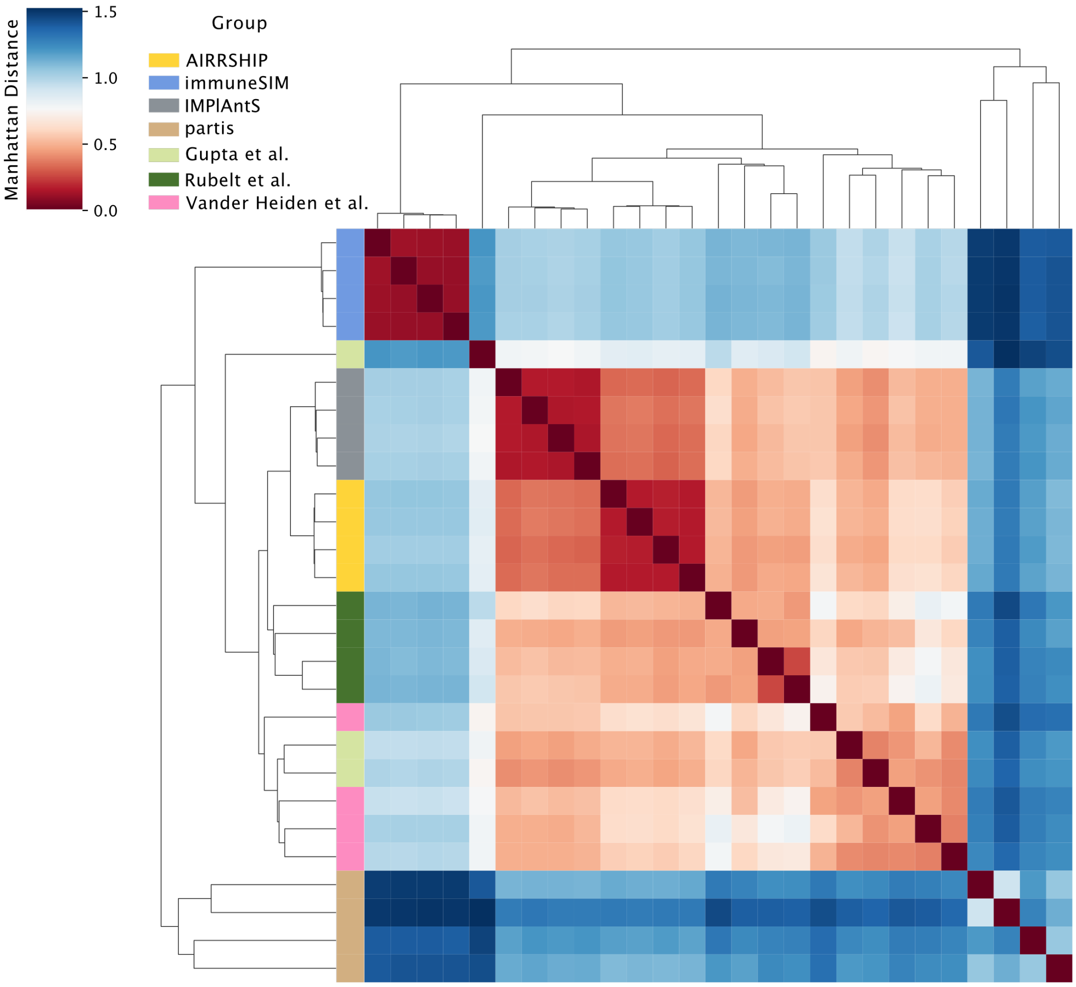

Supplementary Figure S3: Clustermap of Manhattan distance matrix of unique IGHV/IGHJ gene combinations. Individual repertoires (subsampled to 10,000 sequences if larger) from four simulation tools and three 5'RACE experimental datasets were compared. Dataset is indicated by colour of the y-axis. A smaller distance (red) indicates greater similarity between IGHV/IGHJ gene usage, with a distance of 0 indicating identical usage of IGHV/IGHJ gene combinations.

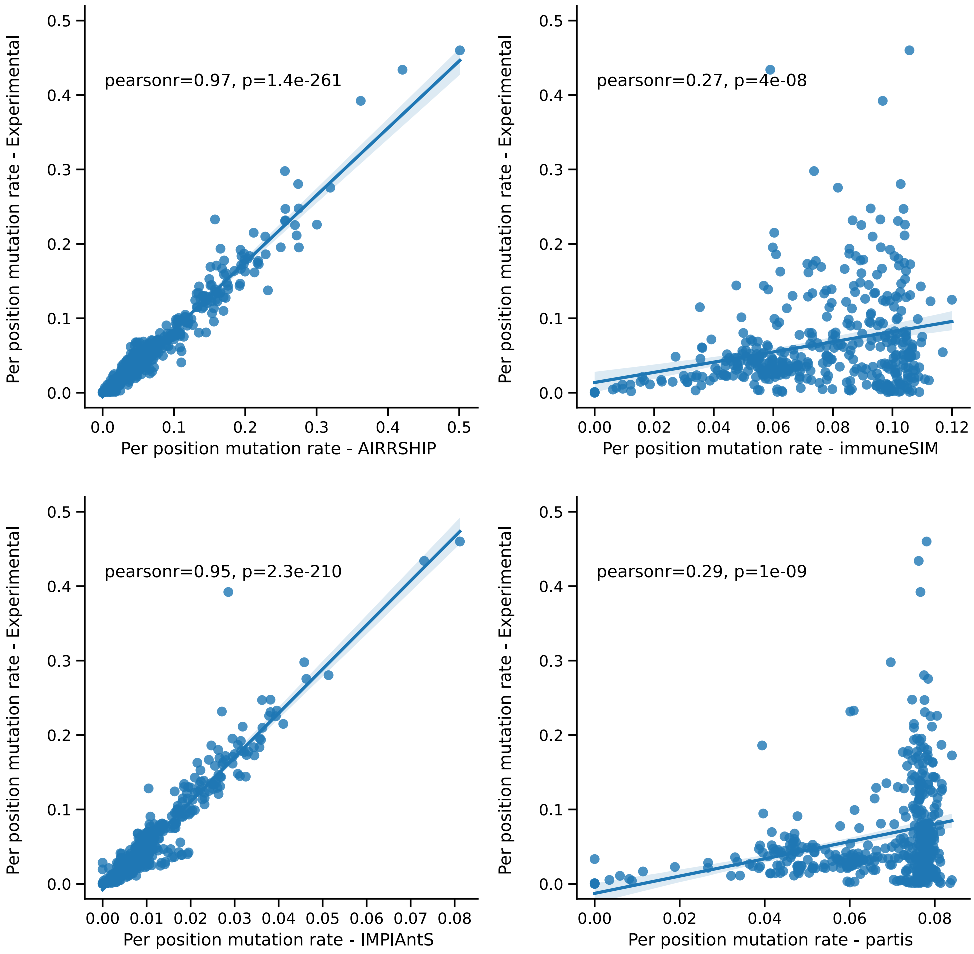

Supplementary Figure S4: Correlation of per position mutation rates across sequences between experimental and simulated sequences from AIRRSHIP, immuneSIM, IMPIAntS and partis. For each tool, four simulated repertoires were combined and compared to the entire set of experimental sequences. Each point represents a single position in the sequence as defined by IMGT unique numbering. Only positions that occurred in at least 1% of sequences were included for each comparison.

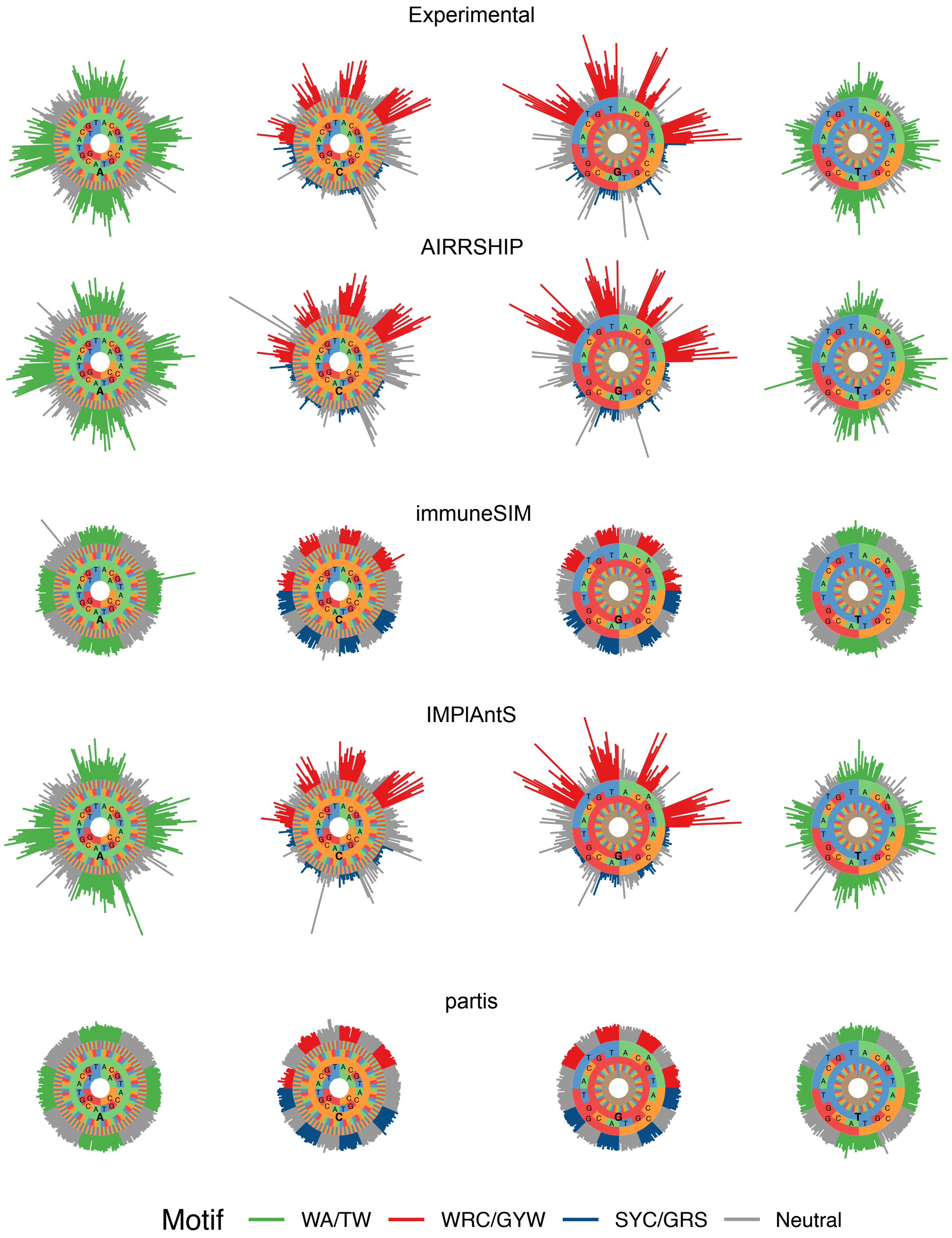

Supplementary Figure S5: Motif mutabilities in experimental data and simulated data. Data are represented as hedgehog plots. All mutations (replacement and synonymous) were analysed using SHazaM. Central concentric circles show the sequence and the length of the bars on the plot of mutability rates corresponds to the likelihood of a given base in the given 5-mer being mutated. Mutability for each kmer was normalised by mean motif mutability for that dataset. The colour of the bars reflects the motif identity (red/green: hotspots, blue: coldspots, and grey: neutral). R = (A or G), S = (C or G), Y = (C or T), and W = (A or T).

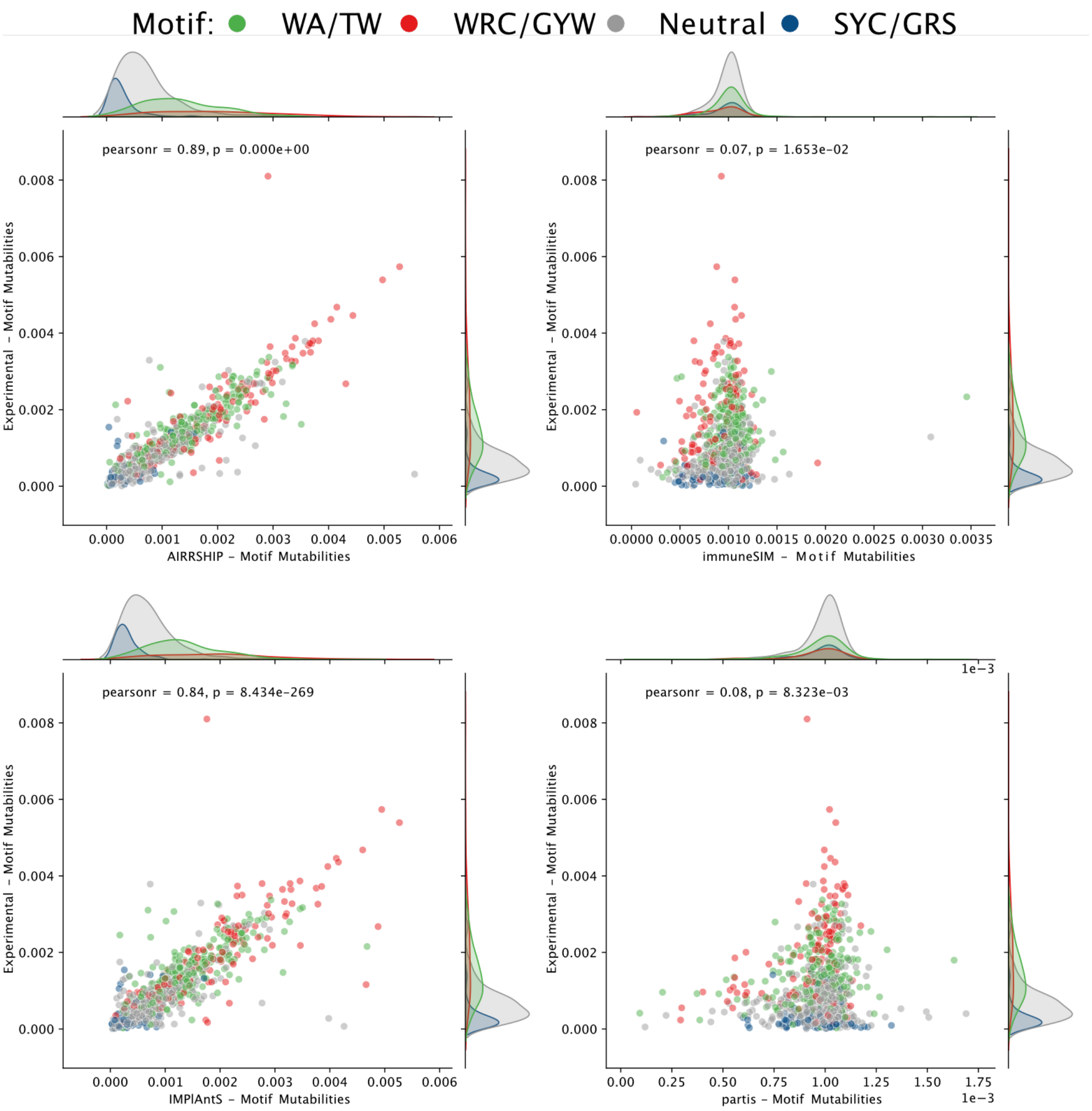

Supplementary Figure S6: Correlation of motif mutability between experimental and simulated sequences. For each tool, four simulated repertoires were combined and compared to the entire set of experimental sequences. Each point represents a single unique 5-mer motif. All mutations (replacement and synonymous) were analysed using SHazaM. Colour of points reflects the motif identity (red/green: hotspots, blue: coldspots, and grey: neutral). R = (A or G), S = (C or G), Y = (C or T), and W = (A or T).

Supplementary Table S5: Runtimes for creation of 10,000 unique VDJ rearrangements, with and without somatic hypermutation. Runtime was measured across ten simulations using either the shell time command or the R system.time() function and is displayed as mean (range). Partis was run using eight processes, all other tools were run as default as no thread specification was possible.

Tool	Mean real time (seconds)	Mean user time (seconds)	Mean real time – SHM (seconds)	Mean user time – SHM (seconds)
AIRRSHIP	7.7 (7.5 – 7.8)	7.6 (7.5 – 7.8)	65.5 (62.4 – 68.5)	64.5 (62.2 – 67.3)
immuneSIM	606.4 (598.4 – 618.1)	606.1 (598.1 – 617.8)	3561.4 (3494.1 – 3622.8)	3559.2 (3492.5 – 3621.2)
IMPlAntS	30.4 (30.2 – 30.5)	30.5 (30.3 – 30.7)	135.8 (133.7 – 137.8)	135.6 (133.5 – 137.5)
partis	100.4 (99.9 – 101.1)	512.0 (510.5 – 513.7)	133.2 (131.5 – 135.9)	691.1 (685.9 – 703.7)

Supplementary Table S6: Maximum memory used when simulating 10,000 unique VDJ rearrangements, with and without somatic hypermutation. Memory use was measured across five simulations using the GNU time command and is displayed as mean (range).

Tool	Mean maximum memory usage (kB)	Mean maximum memory usage – SHM (kB)
AIRRSHIP	21064 (21060 – 21068)	21166 (21164 – 21168)
immuneSIM	466136 (460216 – 468092)	484627 (483748 – 485788)
IMPlAntS	53563 (53560 – 53564)	116134 (115668 – 116500)
partis	37298 (37244 – 37364)	37493 (37408 – 37548)

Supplementary Table S7: Runtimes for creation of 10 - 1,000,000 unique VDJ rearrangements, with and without somatic hypermutation. Runtime was measured across ten or five simulations (100,000/1,000,000 sequences only) using the shell time or the R system.time() command and is displayed as mean (range). Partis was run using eight processes, all other tools were run as default as no thread specification was possible.

**Tool**	**Number of sequences**	**Mean real time (seconds)**	**Mean real time – SHM (seconds)**
AIRRSHIP	10	0.3 (0.3 - 0.3)	0.3 (0.3 - 0.4)
	100	0.3 (0.3 - 0.3)	0.9 (0.8 - 1.1)
	1000	1.0 (1.0 - 1.1)	6.6 (6.3 - 6.7)
	10000	7.7 (7.5 - 7.8)	65.5 (62.4 - 68.5)
	100000	77.0 (75.5 – 78.0)	658.7 (654.4 – 661.6)
	1000000	745.5 (737.6 – 755.5)	6495.8 (6402.8 - 6644.5)
IMPlAntS	10	0.7 (0.7 - 0.7)	3.4 (3.3 - 3.4)
	100	0.9 (0.9 - 0.9)	4.5 (4.3 - 4.7)
	1000	3.6 (3.5 - 3.6)	16.3 (15.7 - 16.9)
	10000	30.4 (30.2 - 30.5)	135.8 (133.7 - 137.8)
immuneSIM	10	2.0 (1.7 - 3.3)	5.3 (3.4 - 7.3)
	100	7.2 (6.6 - 8.1)	35.4 (29.9 - 40.6)
	1000	61.9 (60.2 - 62.8)	346.3 (320.1 - 358.7)
	10000	606.4 (598.4 - 618.1)	3561.4 (3494.1 - 3622.8)
partis	10	3.1 (3.0 - 3.3)	3.3 (3.2 - 3.4)
	100	4.2 (4.0 - 4.3)	4.6 (4.4 - 5.1)
	1000	12.9 (12.8 - 13.3)	16.3 (15.8 - 16.7)
	10000	100.4 (99.9 - 101.1)	133.2 (131.5 - 135.9)

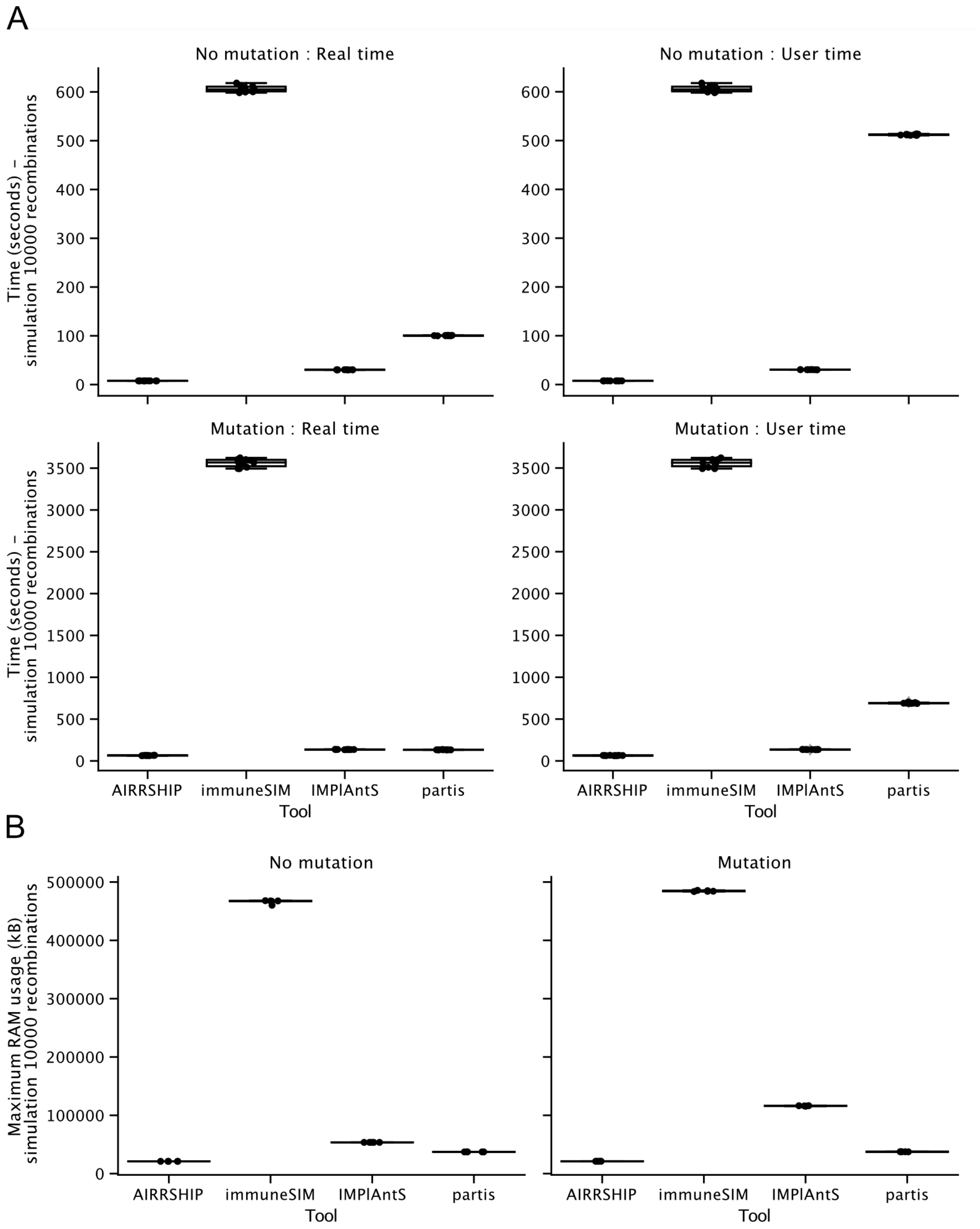

Supplementary Figure S7: A) Runtimes B) Maximum RAM usage for simulation of 10,000 VDJ recombinations for each simulation tool, with and without the introduction of somatic hypermutation. Time was measured using the shell time command, RAM usage was measured using the GNU time command. Real time is equivalent to wall clock time, user time indicates the CPU time involved in the process. These differ only substantially for partis which was run using eight threads.

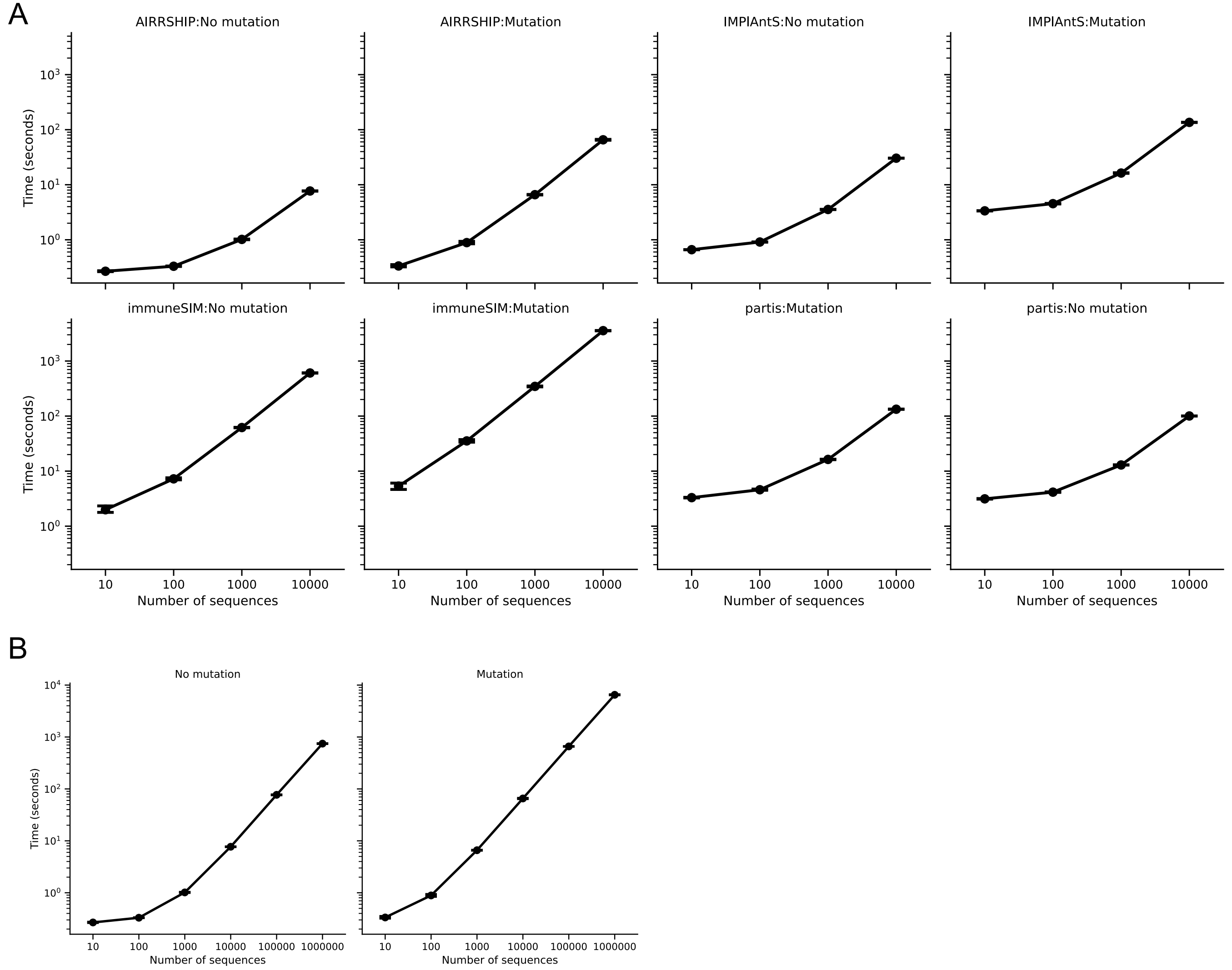

Supplementary Figure S8: A) Wall clock time for simulation of 10 - 10,000 unique VDJ recombinations for each simulation tool. partis which was run using eight threads. B) Wall clock time for simulation of 10 - 1,000,000 VDJ recombinations using AIRRSHIP.

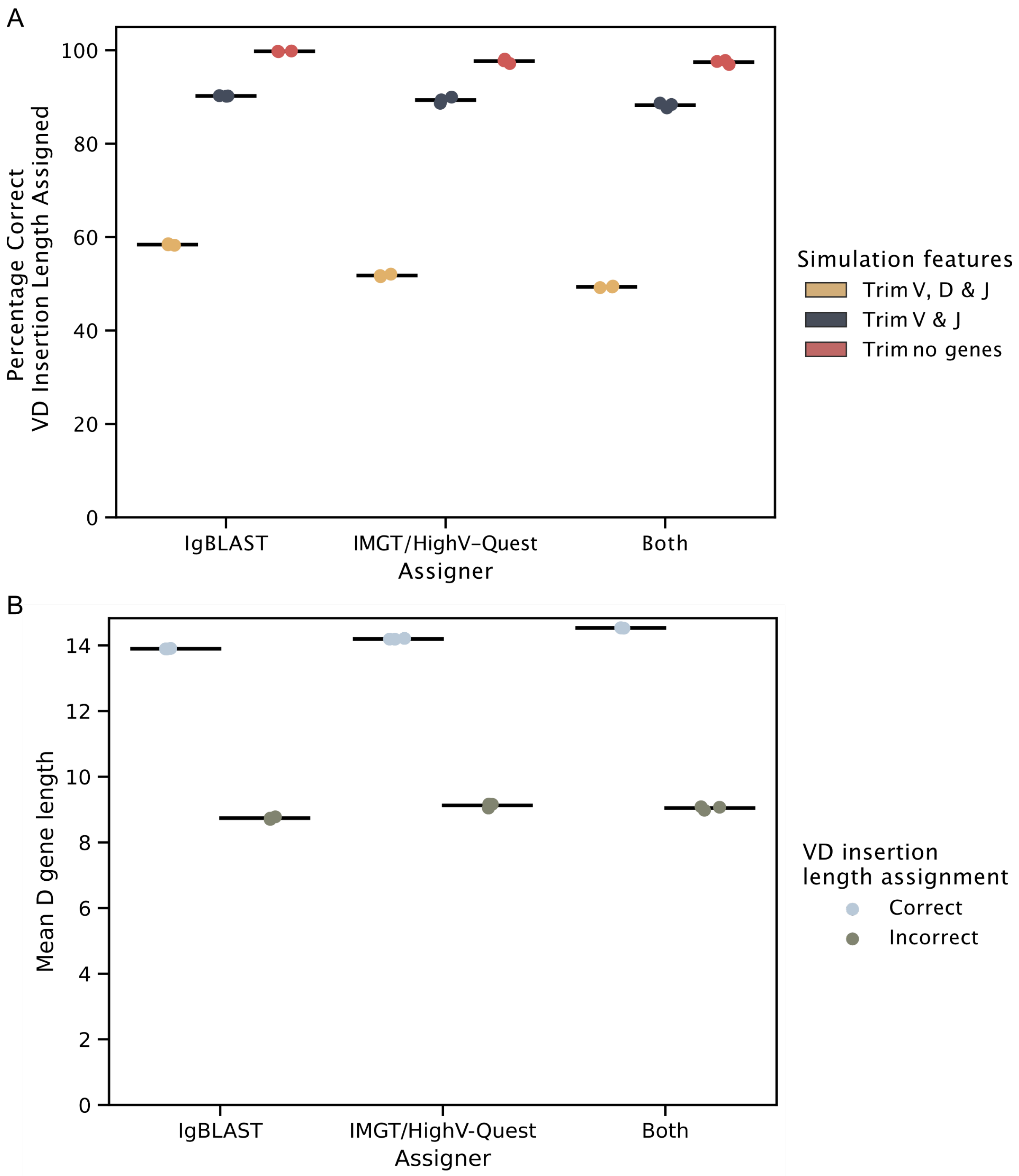

Supplementary Figure S9: A) Percentage of sequences with VD insertion length correctly assigned by IgBLAST, IMGT/HighV-QUEST or both tools when run on repertoires simulated by AIRRSHIP using different trimming parameters. B) Mean true D gene length as recorded by AIRRSHIP when the VD insertion length is correctly or incorrectly assigned.

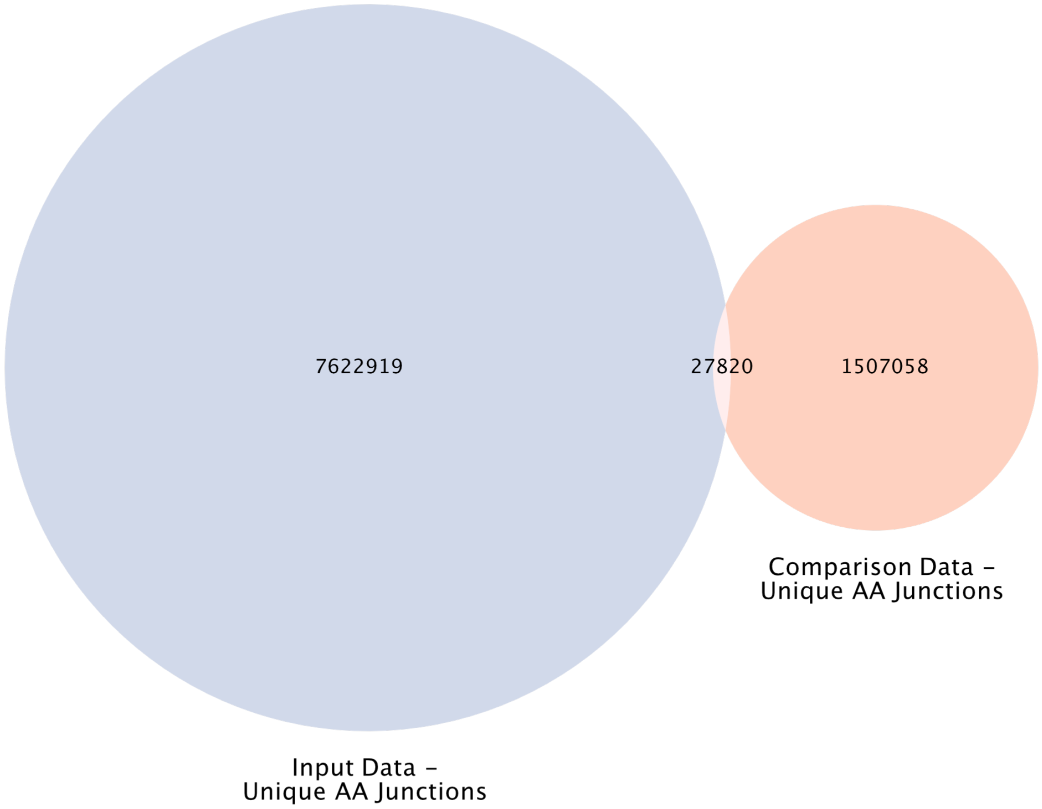

Supplementary Figure S10: Venn diagram showing number of unique amino acid junction sequences present only in the complete input dataset, only in the complete comparison dataset, and those shared between them.
